# Dosimetric validation for an automatic brain metastases planning software using single‐isocenter dynamic conformal arcs

**DOI:** 10.1120/jacmp.v17i5.6320

**Published:** 2016-09-08

**Authors:** Haisong Liu, Jun Li, Evangelos Pappas, David Andrews, James Evans, Maria Werner‐Wasik, Yan Yu, Adam Dicker, Wenyin Shi

**Affiliations:** ^1^ Department of Radiation Oncology Sidney Kimmel Medical College at Thomas Jefferson University Philadelphia PA USA; ^2^ Department of Medical Radiologic Technology Technological Educational Institute of Athens Athens Greece; ^3^ Department of Neurosurgery Sidney Kimmel Medical College at Thomas Jefferson University Philadelphia PA USA

**Keywords:** stereotactic radiosurgery, multiple brain metastases, dosimetry, validation, single isocenter

## Abstract

An automatic brain‐metastases planning (ABMP) software has been installed in our institution. It is dedicated for treating multiple brain metastases with radiosurgery on linear accelerators (linacs) using a single‐setup isocenter with noncoplanar dynamic conformal arcs. This study is to validate the calculated absolute dose and dose distribution of ABMP. Three types of measurements were performed to validate the planning software: 1, dual micro ion chambers were used with an acrylic phantom to measure the absolute dose; 2, a 3D cylindrical phantom with dual diode array was used to evaluate 2D dose distribution and point dose for smaller targets; and 3, a 3D pseudo‐*in vivo* patient‐specific phantom filled with polymer gels was used to evaluate the accuracy of 3D dose distribution and radiation delivery. Micro chamber measurement of two targets (volumes of 1.2 cc and 0.9 cc, respectively) showed that the percentage differences of the absolute dose at both targets were less than 1%. Averaged GI passing rate of five different plans measured with the diode array phantom was above 98%, using criteria of 3% dose difference, 1 mm distance to agreement (DTA), and 10% low‐dose threshold. 3D gel phantom measurement results demonstrated a 3D displacement of nine targets of 0.7±0.4 mm (range 0.2 ~ 1.1 mm). The averaged two‐dimensional (2D) GI passing rate for several region of interests (ROI) on axial slices that encompass each one of the nine targets was above 98% (5% dose difference, 2 mm DTA, and 10% low‐dose threshold). Measured $D_{95}$, the minimum dose that covers 95% of the target volume, of the nine targets was 0.7% less than the calculated $D_{95}$. Three different types of dosimetric verification methods were used and proved the dose calculation of the new automatic brain metastases planning (ABMP) software was clinical acceptable. The 3D pseudo‐*in vivo* patient‐specific gel phantom test also served as an end‐to‐end test for validating not only the dose calculation, but the treatment delivery accuracy as well.

PACS number(s): 87.53.Lv, 87.55.km, 87.55.Qr

## I. INTRODUCTION

Brain metastases represent the most frequent brain tumor and are a significant cause of morbidity and mortality. Surgery, whole‐brain radiation treatment (WBRT), and stereotactic radiosurgery (SRS) are all used in the treatment of brain metastases.^1^, ^2^ Radiosurgery has emerged as a common treatment modality for brain metastases.^3^, ^4^, ^5^ GammaKnife (Elekta AB, Stockholm, Sweden), CyberKnife (Accuray Inc., Sunnyvale, CA), and linear accelerator (linac)‐based SRS are all widely used.

The technique of linac‐based SRS for a single lesion includes circular arcs with stereotactic cones, dynamic conformal arcs (DCA) with multileaf collimator (MLC), intensity‐modulated radiosurgery (IMRS), and volumetric‐modulated arc radiosurgery (VMAS) with the target positioned at linac isocenter. The planning effort and delivery time of this technique is proportional to the number of lesions when treating multiple brain metastases with SRS. The treatment time for one lesion typically ranges from 15 ~ 20 min, which becomes the limit factor to treat more than four to five brain metastases in a single session.

Therefore, some studies have proposed treating multiple metastases with a single setup isocenter using volumetric modulated arc therapy (VMAT) technique.^6^, ^7^, ^8^, ^9^ However, the quality assurance and plan‐specific dose verification of a single‐isocenter multimetastases VMAT plan is a challenge to clinical physicists, due to the nature of small target volumes and low resolution detector arrays.

Huang et al.^(10)^ proposed single‐isocenter dynamic conformal arcs (SIDCA) to treat multiple brain metastases, and showed it has similar plan quality as multi‐isocenter dynamic arc plans, lower peripheral dose spread, but worse conformity than VMAT plans.

A commercial planning software using this technique, called automatic brain metastases planning (ABMP) element from Brainlab AG (Feldkirchen, Germany), has been installed in our institution. It uses a preconfigured set of dynamic conformal arcs to treat up to 10 metastases with a single isocenter. It optimizes the weight of each arc to achieve the best conformity for all targets. Planning time for optimization and dose calculation is 2 ~ 4 min, which improves the planning efficiency dramatically in our clinic.

In this paper, we describe three methods used in our clinic to measure the delivered dose and then compare to the calculated dose distribution, as our initial dosimetry validation of the planning software. ABMP element (version 1.0) cannot perform a phantom mapping process, which would transfer the treatment plan on patient anatomy to a phantom and then recalculate the dose distribution in the phantom for dose verification. Therefore, all three methods in this study treat three different phantoms as actual patients, simulate the entire patient treatment workflow (CT, planning, and radiation delivery), and then compare the measurement to calculation. Therefore, the results demonstrate not only the calculation accuracy, but also the accuracy of the treatment machine and image‐guidance (IGRT) system used in our clinic.

## II. MATERIALS AND METHODS

### A. Introduction to the ABMP element planning

ABMP element is a dedicated, automatic brain‐metastases planning software. It is designed to treat multiple targets (up to 10 in current version 1.0) simultaneously with a single setup isocenter using multiple noncoplanar dynamic conformal arcs (DCA). The isocenter location is automatically placed at the center of mass of all planning target volumes (PTVs), and is not adjustable by the planner. The couch angles of the noncoplanar DCA can be preconfigured in planning templates. The start and stop angles of each arc are first set to default values (10° ~ 170° when couch angle ranges from 0° ~ 90° and 190° ~ 350° when couch angle ranges from 270° ~ 360° (per IEC 61217 convention)), and automatically modified during optimization. Two independent arcs can be used per couch angle. Collimator rotation is used to smear out the interleaf radiation leakage from MLC. The MLC leaves are shaped to conform to each individual PTV with an additional margin of up to 1 mm. While as many PTVs as possible should be treated by each arc for efficiency, not all PTVs have to be treated by any given arc, in order to minimize radiation to normal tissue. Each leaf pair is only allowed to treat one PTV at any time. After the automatic assignment of PTVs to each arc, the weights of the arcs are optimized to achieve the best conformity, which is measured by the conformity index (CI), one for each PTV. Because the dose prescriptions to the PTVs are enforced (at least 99.5% of the PTV volume has to receive the prescription dose) during the optimization, the CI is given by the ratio between volume of total tissue around the PTV receiving more than the prescribed dose and the volume of the PTV. If perfect conformity is achieved, the CI equals to 1.

Planning time for optimization and dose calculation is about 2 ~ 4 min, depending on the complexity of the case. ABMP element uses existing commissioned 6 MV beam data in iPlan RT Dose (version 4.5, Brainlab) in our institution. The linac is a Varian TrueBeam STx equipped with high‐definition MLC and a Brainlab ExacTrac stereotactic X‐ray image guidance system with six degrees of freedom (6‐DOF) robotic couch.

### B. Dual micro ion chambers measurement of absolute dose at center of two targets

**Figure 1:**
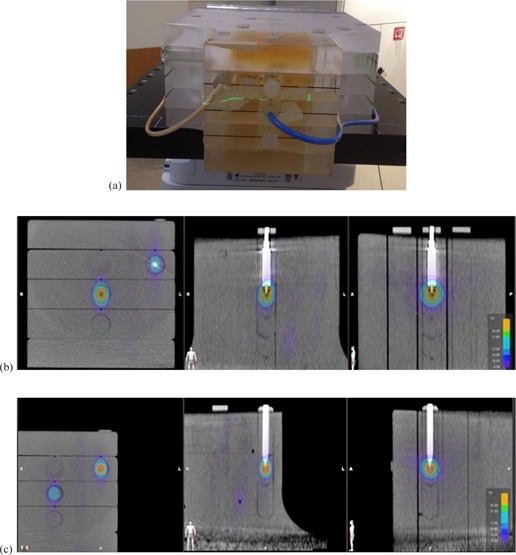
The Standard Imaging acrylic phantom with two microchambers: (a) the setup; (b) three orthogonal CT images centered on the PTW 31014 PinPoint microchamber with isodose overlay; (c) three orthogonal CT images centered on the Standard Imaging A16 microchamber with isodose overlay.

A Standard Imaging (Middleton, WI) acrylic IMRT phantom was used. It has multiple predrilled cavities in the phantom slabs at different locations and plugs with cavities drilled for different types of ion chambers. We used one Standard Imaging A16 microchamber (0.007 cc) and one PTW (PTW‐Freiburg GmbH, Freiburg, Germany) 31014 PinPoint microchamber (0.015 cc) inserted into different slabs, different lateral locations, and to different depths within the phantom, so that the two microchambers were at different coordinates in all three dimensions. Figure 1(a) shows the actual setup of the phantom and two microchambers, and Figs. 1(b) and 1(1) show three orthogonal views of CT images of the phantom centered on each microchamber with isodose overlay.

The phantom was CT‐scanned with both chambers in place. The axial resolution of the CT images is 0.65 mm, and the slice thickness is 1.25 mm. After importing the CT dataset into the planning system, both chambers were identified on CT images, and two small cylindrical targets (1.2 cc and 0.9 cc, respectively) were contoured as planning target volume (PTV) centered at the chamber cavity. PTV #1 was a cylindrical target with 1.1 cm diameter, 1.3 cm length and 1.2 cc volume. PTV #2 was a cylindrical target with 1.0 cm diameter, 1.2 cm length and 0.9 cc volume. The selection of the PTV size is based on the consideration of measurement uncertainty introduced by the microchambers. According to previous studies on small‐field measurement,^11^, ^12^, ^13^ small‐field output correction factors need to be applied to measurement for different types of detectors and varied at different field sizes. At the field size selected in this study (∼1.2 cm), the output correction factors for both microchambers are about 1.01,^(12)^ so that they are within our expected tolerance (±3%) from the overall measurement uncertainties including linac daily output fluctuation, perturbation of small radiation fields by the presence of the chamber, and setup uncertainty. For the target diameter between 0.6 cm and 1 cm, the absolute point dose assessment was evaluated by diode, as described in Materials and Methods section C. For the targets less than 0.6 cm diameter, our clinical practice is to add safety margins to make them larger than 0.6 cm.

The electron density of the phantom was overridden to 1.147, according to the specification from the phantom manufacturer. An SRS treatment plan was made using ABMP element with a prescription of 24 Gy to cover both PTVs, with point doses at each target center about 30 Gy. The plan used four couch angles at 50°, 10°, 350°, and 310°. There are total of five arcs, since two arcs were used at the first couch angle. The plan contained five dynamic conformal arcs. Attention was paid to the arc configuration so that no arc plane intersected with chambers' longitudinal axes. Figures 1(b) and (c) show the dose distribution of the two targets overlaid on phantom CT images.

The phantom was then set up with cone‐beam CT (CBCT) image guidance and irradiated according to the treatment plan. The doses to both chambers were measured and compared to the plan calculation for each arc. The percentage difference between the measured and calculated dose was calculated, and is presented in Table 1.

**Table 1:** Microchamber measurement results for two targets.

*Target Number*	*1 (measured by PinPoint chamber)*	*2 (measured by A16 chamber)*
Volume (${\text{cm}}^{3}$)	1.2	0.9
Diameter (cm)	1.1	1.0
Length (cm)	1.3	1.2
*Dose (Gy)*		*Dose (Gy)*

*Arc Number*	*Calculated*	*Measured*	*% Diff*.	*Calculated*	*Measured*	*% Diff*.
1	10.86	10.84	−0.2%	10.46	10.45	−0.1%
2	10.85	10.83	−0.2%	10.46	10.46	0.0%
3	3.65	3.74	2.6%	5.21	5.38	3.3%
4	5.29	5.45	3.1%	0.36	0.38	5.2%
5	0.10	0.13	27.1%	4.28	4.36	1.8%
Total	30.74	30.98	0.79%	30.77	31.03	0.84%

### C. Dual diode array measurement of dose distribution and GI passing rate

**Figure 2:**
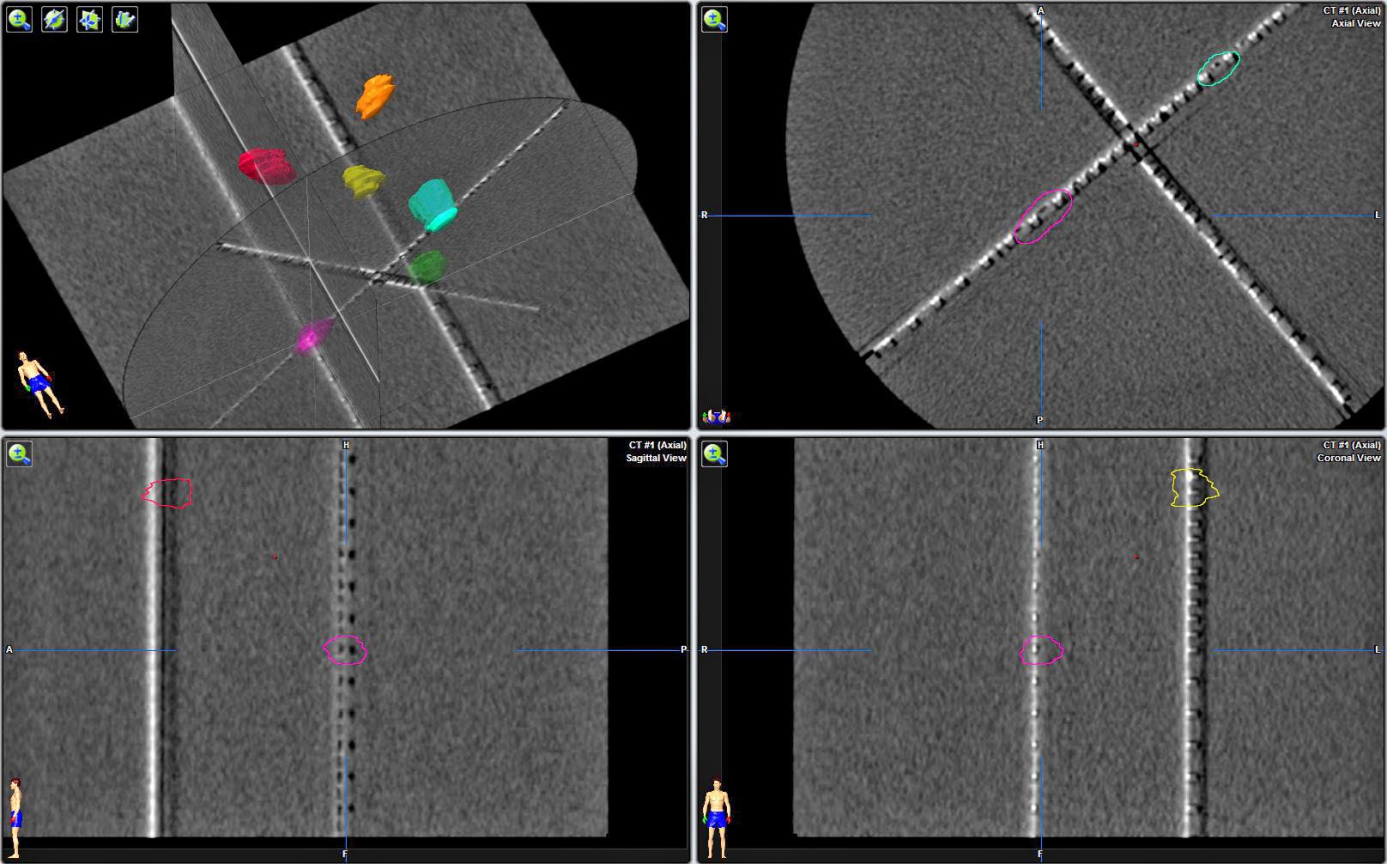
Cross section of CT images of the ${\text{Delta}}^{4}$ phantom with six different targets identified on diode detectors.

The ScandiDos (Uppsala, Sweden) ${\text{Delta}}^{4}$ phantom is a cylindrical phantom made of acrylic, with two embedded orthogonal diode arrays with a total of 1069 diodes. It has a resolution of 2.5 mm in the central region, and 5 mm in the outer region. The phantom was CT scanned and imported into TPS as a patient scan for target contouring and treatment planning. The CT images' axial resolution is 0.51 mm, and slice thickness is 1.25 mm. Electron density of the phantom was overridden to 1.147 according to manufacturer's specification. Figure 2 shows the cross‐section views of the CT image, with six different targets identified on diode detectors.

Five different plans were made using ABMP. All plans have multiple targets, which were all centered around identifiable diodes in the CT dataset. Targets in different plans have different volumes, ranging from 0.1 cc to 2.3 cc (largest linear sizes ranged from 0.6 cm to 3.3 cm).

**Figure 3:**
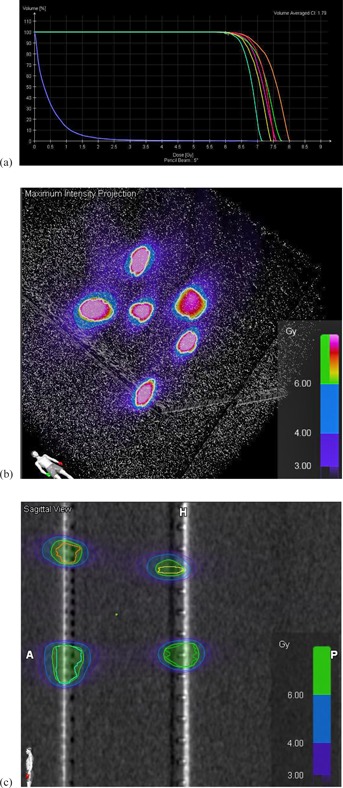
Dose‐volume histogram (DVH) (a) of the plan for the six targets in Fig. 2, all prescribed to 6 Gy. Blue line on the left is the DVH of the entire phantom. 3D dose clouds (b) of the treatment plan; 2D dose distribution (c) on a sagittal plane of the treatment plan.

Table 2 lists the number of arcs and couch angles used in each plan. Since the ${\text{Delta}}^{4}$ phantom cannot measure radiation beams from couch angles larger than $\pm 30^{\circ }$, the couch angles used in these plans were limited accordingly (0° ~ 30° and 330° ~ 359°). Figure 3 shows the dose‐volume histogram (DVH), 3D dose clouds, and 2D dose distribution on a sagittal plane of the plan treating the six targets shown in Fig. 2. The prescription doses to all targets were 6 Gy in this plan. The dose was scaled down from a typical SRS dose to reduce the measurement time.

The ${\text{Delta}}^{4}$ phantom was set up to each plan's isocenter with CBCT guidance, and then irradiated according to the treatment plan. The measured doses at each target center's diodes were compared with the plan calculation. The percentage differences were calculated for each target. The measured and calculated dose profiles across each target were visually examined. The GI passing rate of each plan was calculated using the criteria of 3% dose difference, 1 mm distance‐to‐agreement (DTA), and 10% low‐dose threshold, which is used at our institution for routine VMAS SRS QA. The results are presented in Table 2.

**Table 2:** ${\text{Delta}}^{4}$ Phantom measurement results for five different plans.

*Plans*	*Number of Targets*	*Target Volumes* (${\text{cm}}^{3}$)	*Target Largest Linear Size (cm)*	*Prescription Doses (Gy)*	*Number of Arcs*	*Couch Angles (IEC conventions)*	*Ave. % Dose Difference of Central Diodes (%)*	*Gamma Index (GI) Passing Rate (%)*	*No. of Diodes Above 10% Dose Threshold*
1	4	1.5 ~ 1.55	1.47 ~1.49	6	2	0, 10	−0.6	98.6	216
2	8	0.2 ~ 0.4	0.75 ~ 1.0	4	5	0, 25, 359, 335=2 ^a^	−2.7	98.7	298
3	9	0.1 ~ 0.25	0.6 ~ 0.8	4	7	30×2 ^2^, 10×2 ^a^, $350\times^{2}$ ^a^, 330	−1.8	96.1	258
4	9	0.1 ~ 0.45	0.63 ~ 1.0	4	9	0, 30, 15×2 ^a^, 358, 344×2 ^a^, 330×2 ^a^	0.1	100	351
5	6	1.0 ~ 2.3	1.71 ~ 3.3	6	6	0×2 ^a^, 30×2 ^a^, 345, 330	1.8	99.8	430

^a^ ×2=two arcs at this couch angle.

### D. Pseudo‐*in vivo* patient‐specific phantom filled with polymer gel dosimeter

Polymer gels have been well studied for their dosimetric characteristics.^14^, ^15^, ^16^ In this study, Vinylpyrrolidone‐based polymer gels were used. A detailed description of Vinylpyrrolidone‐(VIPAR) based polymer gels, the manufacturing process, and their dosimetric characteristics can be found in the literature.^17^, ^18^, ^19^, ^20^ Polymer‐gel MRI dosimetry is based on the radiation‐induced polymerization of the gel monomers and cross‐linkers. The amount of absorbed dose in an elementary gel volume is directly related with the amount of polymerization within that volume, and this in turn is directly related to the spin‐spin ($T_{2}$) relaxation time of that volume. An MRI scan of the irradiated polymer gel phantom, at a high spatial resolution can derive a full 3D $T_{2}$ map, which is then converted to a dose map through a calibration process. Moreover, for most polymer gel compositions, a linear dose versus the reciprocal of $T_{2}$ response is observed up to a certain dose level. The polymer gels used in this study were calibrated using a 6 MV X‐ray beam in a dose range of 0 ~ 30 Gy.

A head CT scan of an anonymized patient was sent to RTsafe (RTsafe, PC, Athens, Greece). A hollow phantom that duplicates the patient's skull and internal anatomical bony structures was constructed using a 3D printer, and then filled with polymer gel dosimeter material. Immediately after preparation of the gel and while it is still hot, the gel is poured into the hollow phantom. The gel solidifies at room temperature. Immediately after the production at RTsafe, the gel phantom and six reference vials were placed within a specially designed container that controls its internal temperature range to between 8° and 18° C (melting temperature of the material is 36° C). The polymer gel in the phantom and the gel in the vials are from the same production batch, and experience the same thermal history, from production, through shipment, storage at room temperature in our institution, irradiation, and MRI scanning. The shipment time from RTsafe to us was about two to three working days.

An SRS plan treating nine brain metastases was developed on the CT scan of the phantom. Axial resolution of CT images was 0.59 mm, and slice thickness was 1.25 mm. The volumes of nine targets ranged from 0.11 cc to 0.45 cc, diameters ranged from 0.63 cm to 0.99 cm. They were all planned with 24 Gy to cover 99.5% of the target, with central doses about 30 Gy. Six different couch angles and nine arcs were used in this plan (74°, 42°, 10°, 350°, 318°, and 286°, 2 arcs per couch angle for the last three angles). The phantom was then irradiated as if treating an actual patient. Brainlab ExacTrac stereoscopic X‐ray patient positioning system and a 6‐DOF robotic couch were used to set up the phantom. CBCT was used only at 0° couch angle to confirm the initial setup position. ExacTrac X‐ray imaging was also used for position verification before the radiation delivery at each different couch angle. A repositioning and reverification was performed if shifts and rotations are out of tolerance, which was 0.5 mm for shifts and 0.5° for rotations.

**Figure 4:**
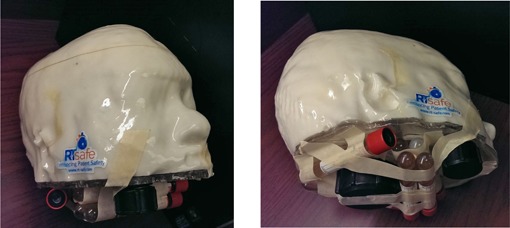
Pseudo‐*in vivo* patient‐specific phantom filled with polymer gel dosimeter. Six vials of gel dosimeters from the same production batch were irradiated to 0, 6, 12, 18, 24, and 30 Gy and then taped onto the phantom in order to obtain MRI scan data simultaneously. They were used as references to crosscheck for RTsafe's dose‐response relationship.

Five vials of gel dosimeters were also irradiated during the same radiation session, with one vial saved for zero‐radiation. The five vials were placed on 10 cm solid water phantom slabs and irradiated to 6 Gy using a 6 MV beam and 10×10 field size with 1.5 cm buildup bolus overlaid on top; then one vial was removed and the remaining vials were given another 6 Gy; repeating this process, the five vials were irradiated to 6, 12, 18, 24, and 30 Gy, respectively. All six vials were then taped onto the phantom so that they can be MRI scanned together (see Fig. 4). They were used as a reference to cross‐check RTsafe's gel response versus the dose relationship.

An MRI scan was acquired 24 hours postirradiation using a GE Optima 1.5T scanner (GE Healthcare, Waukesha, WI). The pulse sequence results in $T_{2}$ maps of the scanned volume. The MRI images were DICOM‐transferred to RTsafe for postprocessing, which converted the $T_{2}$ maps of the 3D MRI scan of the phantom into 3D‐dose distribution measurements. RTsafe did not use the institutions' reference vials to convert MRI $T_{2}$ maps to absolute doses. Therefore, all dosimetry results provided to end‐users are in relative dose.

Several qualitative and quantitative comparisons between calculated and measured dose distributions were provided. A number of dose profiles across treated targets were presented.

One‐dimensional GI calculations along the profile were included with passing criteria of 5% dose difference, 2 mm DTA, and 10% low‐dose threshold. Several rectangular regions of interest (ROIs) encompassing treated targets on axial images were selected for 2D GI calculation. GI passing rates were calculated and presented as color maps. Comparisons of measured and calculated 2D isodose distributions were also presented. Pixels that were too close to the bone/gel interfaces were excluded from the GI calculations since the volume‐averaging effect of a pixel that includes gel and bone material would result in erroneous measurements.

Postprocessed MRI images were also DICOM‐transferred to our institution. They were coregistered with the planning CT images using the automatic fusion function in iPLAN RT Image (version 4.1), which uses a mutual‐information algorithm to fuse images together based on anatomical structures common to both image sets. After image fusion, the contours of original planning target volumes can be overlaid on the post‐irradiation MRI images in which the irradiated volumes are visible, since they present in much different intensities than the unirradiated gel background. Therefore, they are easily segmented out by utilizing the autosegmentation tool in iPLAN RT Image with proper bounding boxes and upper and lower intensity thresholds. After the irradiated volumes were segmented, the central coordinates of the irradiated volumes and those of the planning target volumes were found (center of mass) and thus the displacements were calculated. Results are presented in Table 3.

**Table 3:** Target properties in the treatment plan that was delivered to the gel phantom and the displacements of each target in each direction and in 3D measured from the postirradiation MRI images. Comparisons of the mean dose and $D_{95}$ of all targets are also listed.

*Target*				*Displacements*						*Dose Comparison*		
*Number*	*Volume (cc)*	*Diameter (cm)*	*Distance to Isocenter (cm)*	*Left‐Right (mm)*	*Ant.— Post. (mm)*	*Sup.— Inf. (mm)*	*3D (mm)*	*Mean Dose (%)*		$D_{95}\;(\%)$		
								*TPS*	*Meas*.	*TPS*	*Meas*.	*% Diff*
1	0.11	0.63	0.18	−0.7	−0.1	0.7	0.99	98.3	103.5	95.2	95.7	−0.5%
2	0.26	0.8	4.56	0.6	0.6	−0.6	1.04	99.9	108.9	93.2	92.6	0.7%
3	0.45	0.99	4.42	−0.1	0.1	0.5	0.52	91.9	81.9	96.1	94.9	1.2%
4	0.27	0.84	5.66	−0.1	−0.1	0.1	0.17	100.8	106.1	97.3	98.2	−0.9%
5	0.25	0.79	4.97	0.4	0.5	−0.9	1.10	99.4	105.5	95.0	89.2	6.5%
6	0.27	0.81	5.03	−0.6	−0.6	0.5	0.98	98.8	108.0	93.4	92.6	0.8%
7	0.24	0.8	2.61	−0.2	−0.5	0.6	0.81	97.9	106.7	94.0	93.6	0.4%
8	0.22	0.79	4.08	−0.1	−0.1	0.2	0.24	99.9	107.4	95.2	94.8	0.4%
9	0.28	0.85	4.57	−0.4	0.2	0.5	0.67	99.2	106.4	95.5	97.6	−2.1%
Mean	0.26	0.81	4.01	−0.13	0.00	0.18	0.73	98.5	103.8	95.0	94.3	0.7%
Std. Dev.	0.09	0.09	1.66	0.42	0.40	0.56	0.35	2.6	8.4	1.3	2.8	2.4%
Min.	0.11	0.63	0.18	−0.70	−0.60	−0.90	0.17	91.9	81.9	93.2	89.2	−2.1%
Max.	0.45	0.99	5.66	0.60	0.60	0.70	1.10	100.8	108.9	97.3	98.2	6.5%

## III. RESULTS AND DISCUSSION

### A. Dual micro ion chambers measurement of absolute dose at center of two targets

Table 1 shows the measurement results of both microchambers for all five dynamic arcs. Target 1 had a measured total dose of 30.98 Gy, which is 0.79% higher than the calculated dose of 30.74 Gy. Target 2 had a measured total dose of 31.03 Gy, which is 0.84% higher than the calculated dose of 30.77 Gy. Since arc number 5 did not treat Target 1, as a result of the plan optimization, the plan estimated dose and measured dose were only 0.1 Gy and 0.13 Gy, respectively, and are due to MLC leakage.

While micro ion chambers are not as reliable as Farmer‐type ion chambers or scanning chambers for the absolute dose measurement due to their small volume, inferior signal to noise ratio, large stem, and cable effect, their use in this study did allow clinical physicists a certain level of confidence with expected measurement uncertainties. The selection of target sizes and thus radiation beams' field sizes (∼1.5 cm) was too small to use other types of ion chambers, but large enough to tolerate the uncertainties introduced by microchambers. Smaller (0.6 ~ 1.0 cm) targets' absolute dose were validated by the use of diode arrays, as described in next section.

### B. Dual diode array measurement of dose distribution

**Figure 5:**
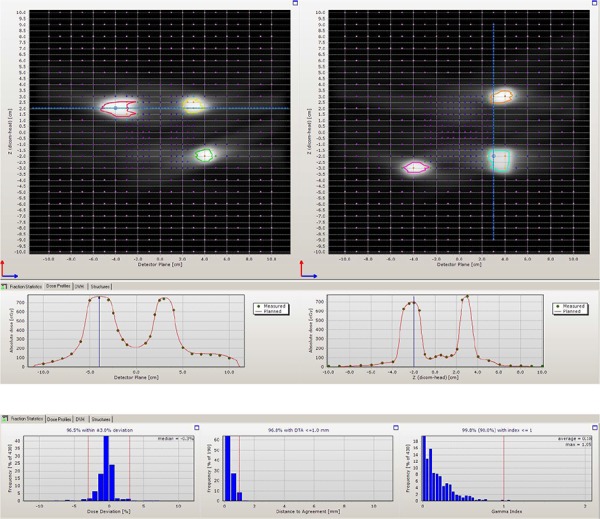
${\text{Delta}}^{4}$ measurement screen capture of plan number 5 with six targets.

Table 2 shows the results of five plans delivered on the ${\text{Delta}}^{4}$ phantom. The averaged dose difference for all 36 targets is −0.8%. The averaged GI passing rate for all plans is 98.6%. Figure 5 shows a screen capture from the ${\text{Delta}}^{4}$ analysis software with irradiated volumes overlaid on two diode detector planes for plan 5. Six targets are clearly seen on two diode planes. The colored contours around each irradiated volume are the contours of the planning targets transferred into ${\text{Delta}}^{4}$ software. The bottom part shows two comparisons of the dose profiles of two selected lines from the detector plane. Continuous lines are the calculated dose profile and the dots are the measured doses from diode detectors. The number of diode detectors above 10% dose threshold and thus used in the GI passing rate calculation were between 216 and 430 points, out of a total of 1069 diodes.

Although the diode array has a poor spatial resolution for finer dose distribution comparison between calculation and measurement, the results obtained from this sampling measurement still provide useful information and confidence regarding the central dose at very small size targets and relative geometric accuracy for each target, as can be seen from Fig. 5. A GI passing rate above 95% from this analysis did give us a very high confidence level on the dose calculation of ABMP element.

### C. Pseudo‐*in vivo* patient‐specific phantom filled with polymer gel dosimeter material

**Figure 6:**
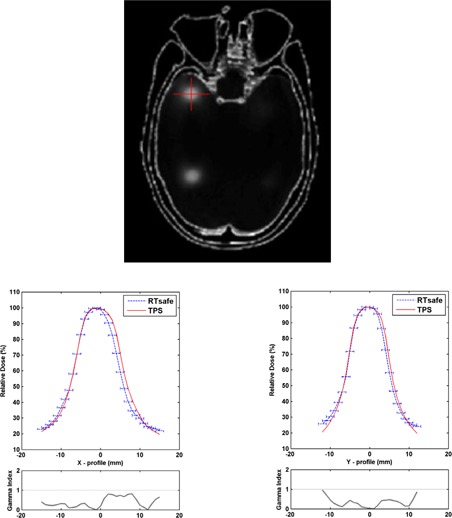
Orthogonal dose profiles comparison at an off‐axis target that is 5.7 cm away from isocenter. 1 mm error bar and 1D gamma index are shown with the profiles.

**Figure 7:**
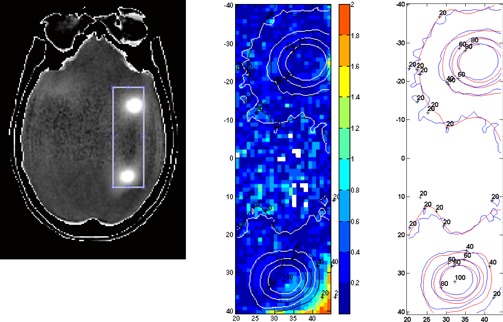
2D GI color map (middle) and 2D isodose lines overlay (right side; blue lines are the RTSafe‐measured dose and red lines are the ABMP‐calculated dose) for the area highlighted on the axial image encompassing two targets (left side). Units are in mm.

Figure 6 shows an orthogonal dose profile comparison of an off‐axis target that was 5.7 cm away from isocenter. The one‐millimeter error bar and 1D gamma index are shown with the profiles. Figure 7 shows 2D GI color maps and overlaid isodose lines for a rectangular ROI of an axial image encompassing two targets. The white pixels in the GI color map had a dose less than 10% and were thus excluded from the GI calculation. The averaged GI passing rate with 5% dose difference, 2 mm DTA, and 10% low‐dose threshold was 98.7% for all ROIs, encompassing all nine targets. Although 1 mm DTA is more preferable to use for SRS plan verification, 2 mm was used in this gel dosimetry study for the consideration of both measurement uncertainties and spatial resolutions ($1\;{\text{mm}}^{3}$ for both dose calculation grid and measured 3D‐MRI dataset). Film dosimetry was an alternative to provide high spatial resolution method for SRS dosimetry. 1D and 2D dose distributions demonstrated using the 3D gel phantom could also be provided by film dosimetry. However, film dosimetry requires proper quality‐controlled equipment and dedicated software for reliable analysis, which were currently not available at our institution.

While a full 3D GI calculation can be obtained from gel phantom dosimetry, RTsafe was still working on their in‐house analysis tool to provide end‐users with that.

**Figure 8:**
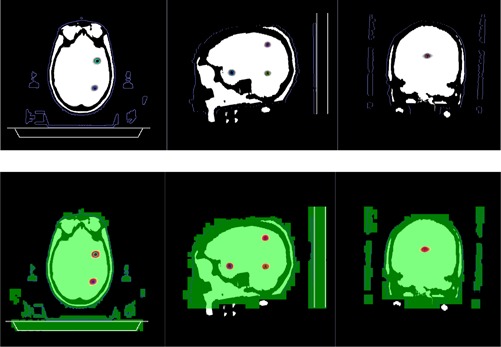
Co‐registration of postirradiation MRI images with planning CT and its associated RTstructures and RTdose calculated by ABMP. Color circles are the planning target volumes. The black areas on the MRI image are the actual irradiated volumes. The red color wash areas are the medium‐high dose regions (<5% relative dose) in the plan.

Figure 8 shows the co‐registration of postirradiation MRI images with planning structures and doses. Color circles are the planning target volumes (PTV). The black areas on the MRI image are the actual irradiated volumes. The red color wash areas are the high‐dose areas in the plan. Brightness and contrast are adjusted so that only high‐dose areas are displayed.

Table 3 lists the volume, diameter, and distance to isocenter of each target, and the displacements in each orthogonal direction and in 3D. The 3D displacement was 0.7±0.4 mm, range from 0.2 to 1.1 mm. These displacements were due to the setup and delivery uncertainties during irradiation and co‐registration uncertainty during the image fusion process. These displacements were within the overall clinical acceptable tolerance for SRS procedures. Table 3 also lists comparison of the mean dose and $D_{95}$ (minimum dose that covers 95% of the target volume) of the nine targets between measurement and calculation. The percentage difference of $D_{95}$ is 0.7%±2.4%. The largest discrepancy of $D_{95}$ (6.5% lower than expected) was from target number 5, which was the target with the largest 3D displacement, 1.1 mm.

## V. CONCLUSIONS

Three different methods were used to validate the dose calculation of our recently installed planning software, Brainlab ABMP, that treats multiple brain metastases with single setup isocenter.

Absolute dose measurement was performed with dual micro ion chambers for targets about 1 cc in volume (∼1.0 cm in diameter). The percentage differences on both targets were less than 1%, which were within our expected ±3% tolerance considering the measurement uncertainties introduced by microchambers and experiment setup.

For smaller targets down to 0.1 cc volume (∼0.6 cm in diameter), diode array detectors in a 3D cylindrical phantom (ScandiDos ${\text{Delta}}^{4}$) were used to measure and then compare both absolute dose and coarse‐sampled 2D dose distributions with calculations. Gamma index with criteria of 3% dose difference, 1 mm DTA, 10% lower‐dose threshold showed an averaged 98.6% passing rate on five different treatment plans. Absolute dose was on average −0.8% for total of 36 targets.

A pseudo‐*in vivo* patient‐specific 3D phantom with gel dosimeters was planned and irradiated as an end‐to‐end test for the overall dosimetric and geometric uncertainties on the planning and radiation delivery system in our clinic. The analysis showed an average of 0.7 mm 3D displacement and 0.7% difference on $D_{95}$ for nine PTVs. Satisfactory dose distribution agreements were observed with an overall 2D GI passing rate of 98.7% with 5% dose difference, 2 mm DTA, and 10% low‐dose threshold.

The ABMP element was in clinical use after these validations. In the current version of ABMP element (version 1.0), there are no “phantom mapping” tools for clinical physicists to transfer a patient plan to a phantom in order to perform measurements and comparisons. Therefore, we perform no patient‐specific verification for clinical cases. The need to have such capability was brought to the manufacture's attention. We hope it will be available in the near future.

Although we used a patient‐specific geometry for the 3D end‐to‐end test, it's really a generic skull phantom for the purpose of the initial commissioning and validation of the ABMP element. However, this test also demonstrated that a patient‐specific QA measurement method can be used in‐clinic, provided shorter turnaround time can be guaranteed by the gel dosimetry service provider.

## ACKNOWLEDGMENTS

The authors would like to thank their reviewers for considerate and thoughtful comments that made the contents of this manuscript more thorough and solid.

## COPYRIGHT

This work is licensed under a Creative Commons Attribution 3.0 Unported License.

## Supporting information

Supplementary Material
