# Supplementary figures and images for: Dosimetric validation for an automatic brain metastases planning software using single‐isocenter dynamic conformal arcs
Source: J Appl Clin Med Phys. 2016 Sep 8;17(5):142–56. doi: 10.1120/jacmp.v17i5.6320 (PMC5874088; doi:10.1120/jacmp.v17i5.6320)

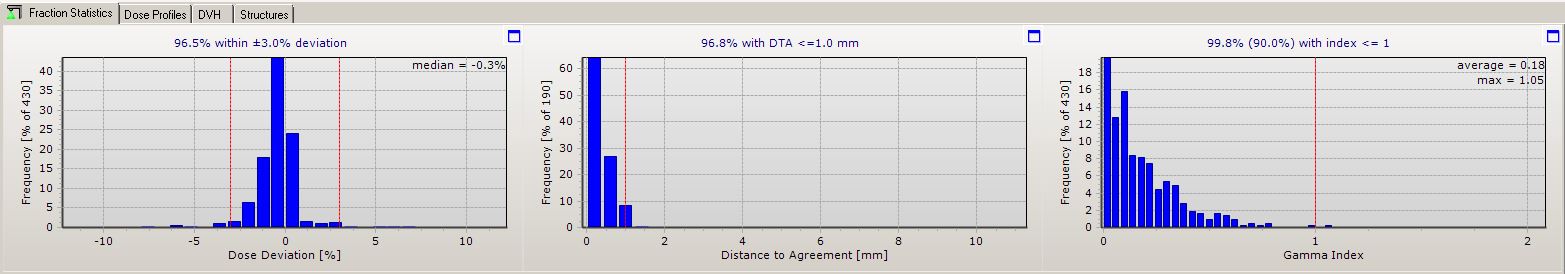

Supplement: Supplementary file 1 — Supplementary Material [file ACM2-17-142-s001.JPG]
